# Emerging Sensor Communication Network-Based AI/ML Driven Intelligent IoT

**DOI:** 10.3390/s23187814

**Published:** 2023-09-12

**Authors:** Bhisham Sharma, Deepika Koundal, Rabie A. Ramadan, Juan M. Corchado

**Affiliations:** 1 Chitkara University Institute of Engineering and Technology, Chitkara University, Rajpura 140401, Punjab, India; 2 Department of Computer Science, University of Petroleum & Energy Studies, Dehradun 248007, Uttarakhand, India; koundal@gmail.com; 3 Computer Engineering Department, College of Computer Science and Engineering, Hail University, Hail 81481, Saudi Arabia; rabie@rabieramadan.org; 4 BISITE Research Group, Edificio Multiusos I+D+i, University of Salamanca, 37007 Salamanca, Spain; jm@corchado.net

At present, the field of the Internet of Things (IoT) is one of the fastest-growing areas in terms of Artificial Intelligence (AI) and Machine Learning (ML) techniques. The utilization of AI and ML is increasingly intertwined with IoT. AI, ML, and deep learning are now being utilized to make IoT services and devices smarter and more secure. Developments in IoT are playing a significant role in our daily lives. In IoT, a large number of devices such as actuators and sensors are being deployed and connected for the collection of different types of data, such as healthcare, transportation, public safety, energy, manufacturing, and smart city infrastructure espousing systems. ML/DL has also shown substantial success in the transformation of complex and massive datasets into precise comprehension as output, which can significantly facilitate intelligence, analysis, automation, and decision-making. ML has provided a way to perform giant modeling and intelligence with the integration of developments in big-data analytics, big-networking technologies, and big-data computing to achieve enormous accomplishments in diverse areas. 

The Special Issue of *Sensors* aims to report on some of the recent innovative studies on advanced techniques in artificial intelligence and machine learning. In [1], the authors proposed an aggregated mutual-information-based feature-selection approach with machine learning methods to enhance the detection of IoT botnet attacks. The NB-IoT benchmark dataset was used to detect botnet attack types using real traffic data gathered from nine commercial IoT devices. The dataset includes binary and multi-class classifications. The feature-selection method incorporates the Mutual Information (MI) technique, Principal Component Analysis (PCA) and ANOVA f-test at a finely granulated detection level to select the relevant features for improving the performance of IoT Botnet classifiers. In the classification step, several ensemble and individual classifiers were used, including Random Forest (RF), XGBoost (XGB), Gaussian Naïve Bayes (GNB), k-Nearest Neighbor (k-NN), Logistic Regression (LR) and Support Vector Machine (SVM). The experimental results showed the efficiency and effectiveness of the proposed approach, which outperformed other techniques using various evaluation metrics. In [2], the authors proposed a security system that was verified by using a real automatic vehicle network dataset, including spoofing, flood, replaying attacks, and benign packets. Preprocessing was applied to convert the categorical data into numerical data. This dataset was processed using the convolution neural network (CNN) and a hybrid network combining CNN and long short-term memory (CNN-LSTM) models to identify attack messages. The results revealed that the model achieved a high performance, as evaluated by the metrics of precision, recall, F1 score, and accuracy. The proposed system achieved high accuracy (97.30%). Along with the empirical demonstration, the proposed system enhanced the detection and classification accuracy compared with the existing systems and was proven to have a superior performance for real-time CAN bus security. Time and frequency domain analyses were performed on the captured data to create feature vectors by selecting the chi-square method, and the most significant features were selected to train the CNN model [3]. The K-means cluster algorithm was used for data-clustering purposes, and the bell curve or normal distribution curve was used to define individual sensor threshold values under normal conditions. The CNN model was used to classify the normal and fault condition data, which provided an accuracy of around 94%, by evaluating the model performance based on recall, precision, and F1 score.

In [4], a Convolutional Neural Network (CNN) is complemented by Transfer Learning to increase the efficiency and accuracy of the early detection of breast cancer for a better diagnosis. The thought process involved the use of a pre-trained model, which already had some weights assigned, rather than building a complete model from scratch. The authors mainly focused on the ResNet101-based Transfer Learning Model paired with the ImageNet dataset. The proposed framework provided us with an accuracy of 99.58%. Extensive experiments and hyperparameter tuning was performed to acquire the best possible results in terms of classification. The proposed frameworks aim to be an efficient tool for all doctors and society as a whole, and to help the user in the early detection of breast cancer. In [5], the performance of the proposed technique was evaluated using a CloudSim toolkit simulator, and the percentage of improvements in the proposed G_SOS over classical SOS and PSO-SA in terms of makespan minimization ranges between 0.61–20.08% and 1.92–25.68% over a large-scale task that spans between 100 and 1000 Million Instructions (MI). The solutions are found to be better than the existing standard (SOS) technique and PSO. The advances in both cyber-attacks and defence strategies are organised in [6]; relevant articles were evaluated to find the impact of the different twists introduced in generic ML/DL algorithms on the vulnerabilities faced by electronic information systems and mobile networks. By providing a brief overview of the most recognised datasets that are utilised to train and test the models, a quality breakdown of the cyber-attack datasets was carried out. Finally, to encourage future researchers and enthusiasts, synopses of current open challenges and potential study areas are laid out. In [7], the authors implemented the RF tree algorithm, which achieved a very low prediction level (MSE = 0.01465) and a correlation R2 (R squared) level of 92.02% with the binary classification dataset, whereas the algorithm attained an R2 level of 89.35% with a multi-classification dataset. The findings of the proposed system were compared with different existing EDoS attack detection systems. The proposed attack mitigation algorithms, which were developed based on artificial intelligence, outperformed the few existing systems. The goal of this research is to enable the detection and effective mitigation of EDoS attacks.

In [8], the authors define three highlighted contributions: first is the enhanced security with blockchain, along with PUFs and duel-gaming-based authentication; second is the ability to use a lightweight blockchain that is compatible with the physical layer, which is termed a branched blockchain, and further evolved to an HLF-oriented local blockchain at layer 2 and Ethereum-based global blockchain at layer 3; third is the ability to deal with physical cloning and side-channel attacks. In [9], the authors presented a comprehensive overview of the latest developments in context-aware intelligent systems using sensor technology. In addition, they discuss the areas in which they are used, related challenges, and motivations to adopt AI solutions, focusing on edge computing, i.e., sensor and AI techniques, along with an analysis of existing research gaps. Another contribution of this study is the use of a semantic-aware approach to extract survey-relevant subjects. The latter specifically identifies 11 main research topics supported by the articles included in the work. These are analysed from various angles to answer five main research questions. In [10] proposed system utilizes the Log of Round value-based Elliptic Curve Cryptography (LR-ECC) to enhance the security level during data transfer after the initial authentication phase. The authorized healthcare staff can securely download the patient’s data on the hospital side. Utilizing the Herding Genetic Algorithm-based Deep Learning Neural Network (EHGA-DLNN) can test these data using the trained system to predict the diseases. The experimental results demonstrate that the proposed approach improves prediction accuracy, privacy, and security compared to the existing methods. In [11], the authors proposed a novel multi-scale feature-fusion of a convolution neural network (CNN) and an improved canny edge algorithm that segregates fracture and healthy bone image. The hybrid scale fracture network (SFNet) is a novel two-scale sequential DL model. This model is highly efficient for bone fracture diagnosis and takes less computational time compared to other state-of-the-art deep CNN models. The innovation of this research is that it works with an improved canny edge algorithm to obtain edges in the images that localize the fracture region. After that, grey images and their corresponding canny edge images are fed to the proposed hybrid SFNet for training and evaluation. Furthermore, the performance is also compared with the state-of-the-art deep CNN models on a bone image dataset. The results showed that SFNet with canny (SFNet + canny) achieved the highest accuracy, F1-score and recall, of 99.12%, 99% and 100%, respectively, for bone fracture diagnosis. This showed that using a canny edge algorithm improves the CNN performance.

In [12], the authors proposed the YOLOv4-TP-Tiny based on the YOLOv4 model, which mainly includes two modules, two-dimensional attention (TA) and pedestrian-based feature extraction (PFM). First, they integrate the TA mechanism into the backbone network, which increases the attention of the network to the visible area of pedestrians and improves the accuracy of pedestrian detection. Then, the PFM is used to replace the original spatial pyramid pooling (SPP) structure in the YOLOv4 to obtain the YOLOv4-TP algorithm, which can adapt to different sizes of people to obtain higher detection accuracy. To maintain this detection speed, we replaced the normal convolution with a ghost network with a TA mechanism, resulting in the appearance more feature maps with fewer parameters. The experimental results show that the YOLOv4-TP-tiny has 58.3% AP and 31 FPS in the winder person pedestrian dataset. When using the same hardware conditions and dataset, the AP of the YOLOv4-tiny is 55.9%, and the FPS is 29. In [13], the authors discuss the challenges and recommendations for the effective implementation of digital technologies in the energy sector to meet the sustainability requirements. The use of big data for energy analytics, digital twins in smart grid modeling, virtual power plants with Metaverse, and green IoT are the major vital recommendations that are discussed in this study for future enhancement. In [14], the authors demonstrate that, out of an initial 25 × 25 population of DNA Keys, 14 keys are rendered weak. Complete results and calculations of how each weak key can be strengthened by the generation of four new populations are illustrated. The analysis of the proposed scheme for different initial populations shows that a maximum of eight new populations must be generated to strengthen all 500 weak keys of a 500 × 500 initial population.

In [15], the authors developed an elaborative stepwise stacked artificial neural network (ESSANN) algorithm to greatly improve automation industries controlling and monitoring the industrial environment. Initially, an industrial dataset provided by KLEEMANN Greece was used. The collected data were then pre-processed. Principal component analysis (PCA) was used to extract features, and feature selection was based on the least absolute shrinkage and selection operator (LASSO). Subsequently, the ESSANN approach is proposed to improve automation industries. The performance of the proposed algorithm was also examined and compared with that of existing algorithms. The key factors compared with existing technologies are delay, network bandwidth, scalability, computation time, packet loss, operational cost, accuracy, precision, recall, and mean absolute error (MAE). Compared to traditional algorithms for industrial automation, the proposed techniques achieved high results, such as a delay of approximately 52%, network bandwidth accomplished at 97%, scalability attained at 96%, computation time acquired at 59 s, packet loss achieved at a minimum level of approximately 53%, an operational cost of approximately 59%, accuracy of 98%, precision of 98.95%, recall of 95.02%, and MAE of 80%. In [16], to examine the performance of the proposed model, two real datasets, namely the Car-Hacking and UNSE-NB15 datasets, were used. Real automated vehicle network datasets were used in the verification process of the proposed security solution. These datasets included spoofing, flooding, and replay attacks, as well as benign packets. The categorical data were transformed into numerical form via pre-processing. Machine learning and deep learning algorithms, namely k-nearest neighbour (KNN) and decision trees, long short-term memory (LSTM), and deep autoencoders, were employed to detect CAN attacks. According to the findings of the experiments, using the decision tree and KNN algorithms as machine learning approaches resulted in accuracy levels of 98.80% and 99%, respectively. On the other hand, the use of LSTM and deep autoencoder algorithms as deep learning approaches resulted in accuracy levels of 96% and 99.98%, respectively. The maximum accuracy was achieved when using the decision tree and deep autoencoder algorithms. Statistical analysis methods were used to analyse the results of the classification algorithms, and the determination coefficient measurement for the deep autoencoder was found to reach a value of R2 = 95%. The performance of all the models that were built in this way surpassed that of those already in use, with almost perfect levels of accuracy being achieved. The developed system can overcome security issues in IVNs. In [17], the authors proposed a route that is determined between the current location of an emergency vehicle and its target location in an emergency. Communication between traffic lights is provided with a mobile application developed specifically for the vehicle driver. In this process, the person controlling the lights can turn on the traffic lights during the passage of vehicles. After the vehicles with priority to pass had passed, traffic signaling was normalized via the mobile application. This process was repeated until the vehicle reached its destination.
